# Validation of protein arginine methyltransferase 5 (PRMT5) as a candidate therapeutic target in the spontaneous canine model of non-Hodgkin lymphoma

**DOI:** 10.1371/journal.pone.0250839

**Published:** 2021-05-14

**Authors:** Shelby L. Sloan, Kyle A. Renaldo, Mackenzie Long, Ji-Hyun Chung, Lindsay E. Courtney, Konstantin Shilo, Youssef Youssef, Sarah Schlotter, Fiona Brown, Brett G. Klamer, Xiaoli Zhang, Ayse S. Yilmaz, Hatice G. Ozer, Victor E. Valli, Kris Vaddi, Peggy Scherle, Lapo Alinari, William C. Kisseberth, Robert A. Baiocchi

**Affiliations:** 1 Department of Veterinary Biosciences, College of Veterinary Medicine, The Ohio State University, Columbus, Ohio, United States of America; 2 Division of Hematology, Department of Internal Medicine, The Ohio State University, Columbus, Ohio, United States of America; 3 Department of Veterinary Clinical Sciences, The Ohio State University, Columbus, Ohio, United States of America; 4 Department of Pathology, The Ohio State University, Columbus, Ohio, United States of America; 5 Department of Biomedical Informatics, Center for Biostatistics, The Ohio State University, Columbus, Ohio, United States of America; 6 VDx Veterinary Diagnostics, Davis, California, United States of America; 7 Prelude Therapeutics, Wilmington, Delaware, United States of America; Colorado State University, UNITED STATES

## Abstract

Non-Hodgkin lymphoma (NHL) is a heterogeneous group of blood cancers arising in lymphoid tissues that commonly effects both humans and dogs. Protein arginine methyltransferase 5 (PRMT5), an enzyme that catalyzes the symmetric di-methylation of arginine residues, is frequently overexpressed and dysregulated in both human solid and hematologic malignancies. In human lymphoma, PRMT5 is a known driver of malignant transformation and oncogenesis, however, the expression and role of PRMT5 in canine lymphoma has not been explored. To explore canine lymphoma as a useful comparison to human lymphoma while validating PRMT5 as a rational therapeutic target in both, we characterized expression patterns of PRMT5 in canine lymphoma tissue microarrays, primary lymphoid biopsies, and canine lymphoma-derived cell lines. The inhibition of PRMT5 led to growth suppression and induction of apoptosis, while selectively decreasing global marks of symmetric dimethylarginine (SDMA) and histone H4 arginine 3 symmetric dimethylation. We performed ATAC-sequencing and gene expression microarrays with pathway enrichment analysis to characterize genome-wide changes in chromatin accessibility and whole-transcriptome changes in canine lymphoma cells lines upon PRMT5 inhibition. This work validates PRMT5 as a promising therapeutic target for canine lymphoma and supports the continued use of the spontaneously occurring canine lymphoma model for the preclinical development of PRMT5 inhibitors for the treatment of human NHL.

## Introduction

Lymphoma is one of the most common malignant cancers in dogs, accounting for 7–24% of all cancers and 83% of hematopoietic malignancies. The majority of lymphomas in dogs are histopathologically high-grade and of B-cell origin. B-cell lymphoma accounts for up to 75% of canine lymphomas [1,2]. Diffuse large B-cell lymphoma (DLBCL) is the most common subtype in both canine and human non-Hodgkin lymphoma (NHL), with the other most commonly occurring subtypes in dogs being nodal marginal zone lymphoma (MZL) and peripheral T-cell lymphoma not otherwise specified (PTCL) with other histologic subtypes being less common [1,3–6]. Molecular classification of DLBCL into activated B-cell (ABC) and germinal center B-cell (GCB) groups is only partially supported in canine lymphoma pathology [6–8]. The current most effective treatment for intermediate/high-grade lymphoma in dogs is multi-agent cytotoxic chemotherapy, with the best remission and survival times being reported with doxorubicin-based sequential chemotherapy protocols similar to CHOP (cyclophosphamide, vincristine, doxorubicin, prednisone) [9,10]. Unfortunately, even using such multi-agent cytotoxic chemotherapy protocols, the median survival time for affected dogs is only about one year, with an approximate 20% two year survival [10].

Likewise, in humans, NHL is a heterogeneous group of blood cancers that arise in hematopoietic tissues including lymph nodes, spleen, and bone marrow. NHL represents 4.6% of all cancer diagnoses in North America, being the fifth most common cancer in women and sixth most common in men [11]. DLBCL is an aggressive malignancy of mature B-lymphocytes, with an annual incidence of just over 25,000 new cases in the United States [5,12]. Standard of care includes a combination of anthracycline-based CHOP chemotherapy in combination with the CD20-specific monoclonal antibody rituximab, and involved field radiation therapy in select cases [13]. Patients with DLBCL have highly variable clinical courses; and, while most patients respond initially to immuno-chemotherapy, fewer than half of patients achieve a durable complete remission [5,14]. There is a need for discovery of new therapeutic targets and development of novel, well-tolerated therapies for this disease, especially in the relapsed, refractory patient population.

Protein arginine methyltransferase 5 (PRMT5) is a type II protein arginine methyltransferase (PRMT) enzyme that is frequently dysregulated in both solid and hematologic cancers [15,16]. PRMTs catalyze the arginine methylation of histone tails and other cellular proteins that modulate a diverse array of cellular processes including transcriptional regulation, RNA processing, cell signaling, differentiation, DNA damage response, apoptosis, and tumorigenesis [15,17]. While both type I and type II PRMTs catalyze mono-methylation at the ω-NH2 of arginine residues, they differ in their ability to add a second methyl group, either asymmetrically (type I) or symmetrically (type II). PRMT5 catalyzes the symmetric di-methylation of histone proteins H2A (H2AR3me2s), H3 (H3R8me2s) and H4 (H4R3me2s), altering chromatin structure to promote transcriptional gene silencing [18–20]. Symmetric dimethyaltion of histone H3 (H3R2me2s) by PRMT5 has been shown to maintain a euchromatic state and support gene expression [21,22]. In addition to histone methylation, PRMT5 is known to methylate a wide variety of proteins including PIWI, the spliceosome Sm, EGFR, E2F1, p53, the p65 subunit of NF-kB, and RAD9, to impact a broad spectrum of cell signaling pathways relevant to cancer biology [15,23,24]. More recently, it has been demonstrated in DLBCL that methylation of BCL6 by PRMT5 is necessary for germinal center formation and affinity maturation [25].

PRMT5 overexpression is involved in cell proliferation and survival in DLBCL and mantle cell lymphoma (MCL), as well as a variety of solid tumors including glioblastoma, lung carcinoma, melanoma, hepatocellular carcinoma (HCC), and colorectal cancer [26–32]. We and others have previously shown PRMT5 to be dysregulated and to contribute to lymphomagenesis in different histologic subtypes, using a variety of experimental *in vivo* model systems of MCL, DLBCL, acute leukemia, and spontaneous models of oncogenic viral-induced lymphoproliferative disease (EBV-LPD) [17,26,27,33,34]. EBV-induced lymphomas and transformed cell lines exhibit abundant expression of PRMT5 critical to EBV-driven B-cell transformation and maintenance of the malignant phenotype [33]. In Eμ-myc transgenic mice, *MYC* directly upregulates PRMT5 and tumor progression is delayed after heterozygous deletion of the *PRMT5* gene [35]. In DLBCL, PRMT5 expression can be induced upon stimulation of the B-cell receptor (BCR) and downstream signaling pathway, while the overexpression of PRMT5 activates PI3K-AKT signaling creating a positive feedback loop to promote cell cycle progression and survival [36]. Additionally, PRMT5 promotes polycomb repressor complex (PRC2) expression through the transcriptional silencing of *RBL2* and hyperphosphorylation of RB1 through enhanced cyclin D1 expression. Both PRMT5 and PRC2 levels are elevated in MCL and DLBCL [37]. In myeloproliferative neoplasms, PRMT5 has been shown to regulate E2F target gene expression by modulating the symmetric dimethylation status of E2F1 [38]. Together these findings demonstrate the multifaceted role of PRMT5 in regulating the growth and survival of lymphoma cells.

While the detailed mechanisms underlying the cell-transforming capabilities of PRMT5 remain unclear, knockdown of PRMT5 expression by genetic or pharmacologic means has been shown to modulate the methylation status of target proteins and exert antitumor activities *in vitro* and *in vivo* [33,39]. Anti-proliferative effects were demonstrated with the selective PRMT5 inhibitor EPZ015666 (GSK3235025) in MCL cell lines and mouse xenografts [33,39]. Knockdown of PRMT5 with siRNA in HCC cells suppressed proliferation and induced cell cycle arrest *in vitro*. Treatment of HCC and colorectal cancer cells *in vitro* and *in vivo* in subcutaneous xenografts with the non-selective PRMT inhibitor AMI-1 inhibited cellular proliferation [31,32]. Our collaborative group has developed a series of first-in-class small molecule inhibitors of PRMT5 (HLCL65, C220, PRT382, PRT543) demonstrating antitumor activity in MCL, DLBCL, HTLV-1 induced adult T-cell leukemia/lymphoma, acute myeloid leukemia (AML), and glioblastoma cells [28,33,34,40,41]. PRT543 demonstrates a high degree of selectivity for PRMT5 and potent anti-tumor activity and is currently being tested in a phase I human clinical trial for the treatment of advanced solid tumors and hematological malignancies (NCT03886831).

Increasingly, clinical trials in dogs with spontaneously occurring lymphoma and other cancers have been incorporated into the preclinical development of anticancer agents [42–48]. To validate canine lymphoma as a useful comparison to human lymphoma while validating PRMT5 as a rational therapeutic target in canine lymphoma, we evaluated the prevalence of PRMT5 expression and correlation with histological subtype in canine lymphomas. We characterized the expression of PRMT5 in canine lymphoma cell lines and primary canine samples and determined the effects of PRMT5 inhibition on canine lymphoma cell proliferation and viability, as well as key biomarkers of PRMT5 activity. Using the Assay for Transposase-Accessible Chromatin with high-throughput sequencing (ATAC-seq), we show a lack of global differential chromatin accessibility after PRMT5 inhibition, while detailing specific sites of chromatic gene silencing relevant to PRMT5 biology including *MYC*. Using a canine transcriptional microarray platform, we profile whole-transcriptome changes induced by inhibition of PRMT5, highlighting the commonalities between human and canine PRMT5 biology. This work validates PRMT5 as a promising therapeutic target for canine lymphoma and supports the continued use of the spontaneously occurring canine lymphoma model for the preclinical development of PRMT5 inhibitors for the treatment of NHL in people.

## Results

### PRMT5 is overexpressed in canine lymphoma subtypes

**Fig 1:**
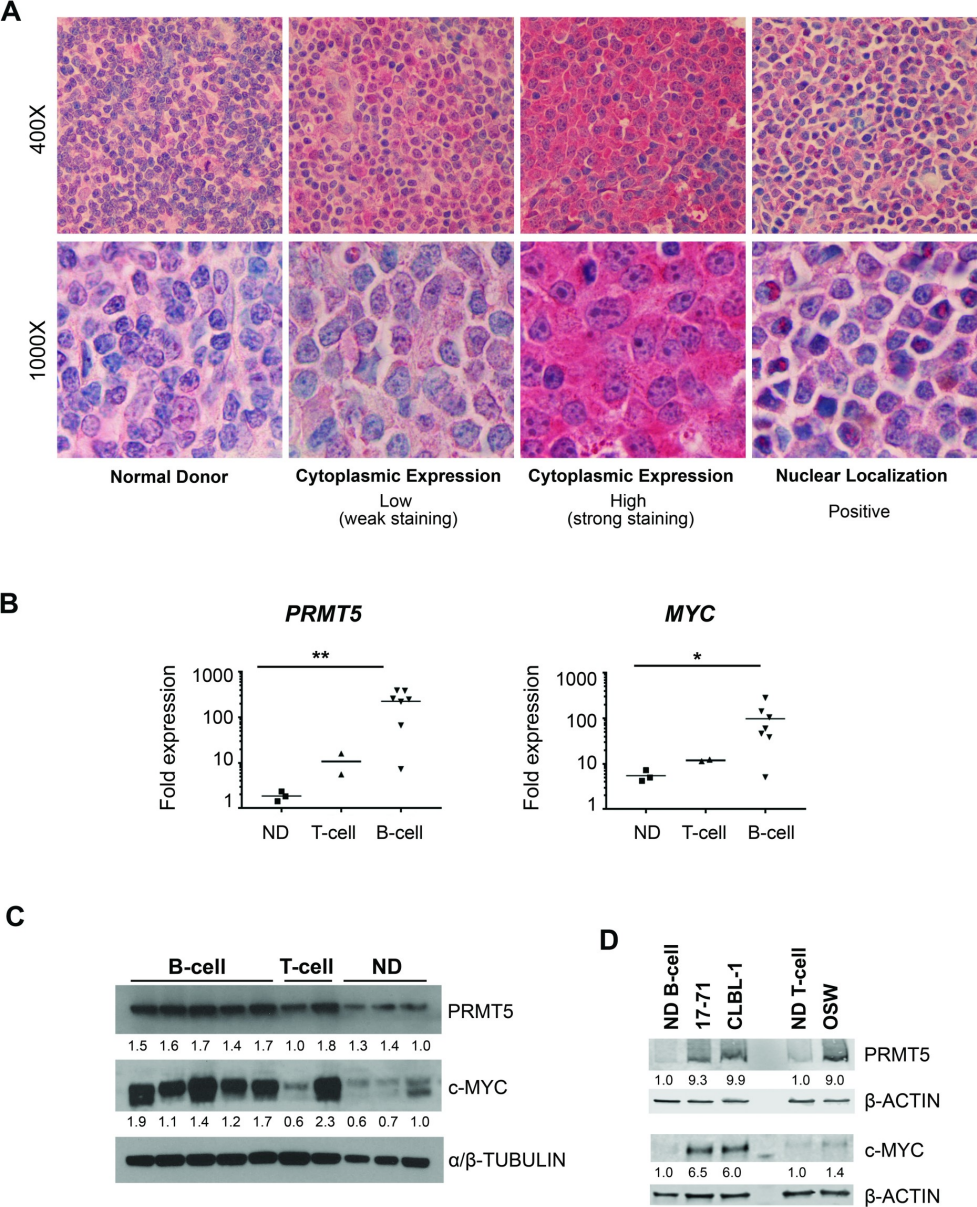
PRMT5 is overexpressed in canine lymphoma subtypes. (A) Photomicrographs of representative histologic sections from primary canine lymphomas (n = 337) and normal donor (ND) lymph node samples (n = 40) corresponding to Table 1. Anti-PRMT5 IHC. Representative ND tissue shows weak cytoplasmic staining with lack of nuclear staining for PRMT5, in comparison to lymphoma samples with either weak cytoplasmic staining, strong cytoplasmic staining, or presence of nuclear staining for PRMT5. (B) Quantitative RT-PCR showing the relative fold change expression of *PRMT5* and *MYC* mRNA in primary canine B-cell (n = 7) and T-cell (n = 2) lymphoid biopsy samples compared to ND samples (n = 3). Dots represent different lymphoid samples from different donors. Statistical significance evaluated using multiple paired two-tailed Student’s t-Test assuming unequal variance. The respective p-values are indicated (*p < 0.*05*, **p < 0.*01*) (C) Immunoblot analysis of PRMT5 and MYC indicate elevated protein expression in primary canine B-cell lymphoma biopsies (n = 5) compared to ND lymphoid tissue. One of two primary T-cell lymphoma specimens showed elevated PRMT5 and MYC protein levels. PRMT5, MYC, and α/β-TUBULIN were ran on separate gels using the same protein lysates. Immunoblots were detected using film and HRP conjugated secondary antibodies. (D) Immunoblot analysis of PRMT5 and MYC in the canine B-cell lymphoma cell lines (17–71 and CLBL-1) and T-cell lymphoma cell line (OSW). Compared to isolated B- and T-lymphoid cells from ND peripheral blood, canine lymphoma cell lines overexpress protein levels of PRMT5 and MYC, respectively. Immunoblots were detected with fluorescently labeled Licor IRDye secondary antibodies. For (C) and (D) protein expression was quantified by the ratio of target protein to loading control and normalized to ND.

Spontaneous lymphoma in dogs shares many histopathological, genetic, and clinical features with NHL in humans. Given the relevance of PRMT5 to lymphomagenesis in humans, we hypothesized that PRMT5 would be overexpressed in canine lymphomas. To assess expression profiles of PRMT5 in canine lymphomas, we performed immunohistochemistry on canine lymphoma tissue micro arrays (TMAs). In control lymph nodes from dogs without cancer (n = 40), PRMT5 immunoreactivity was weak and expressed only in the cytoplasm (Table 1 and Fig 1A). In canine lymphomas of various histopathological subtypes (n = 337), PRMT5 immunoreactivity was more variable in intensity and subcellular localization with strong cytoplasmic expression in 42.40% of samples. Notably, DLBCL, the most common lymphoma in dogs, showed strong cytoplasmic expression in 48.80% of the samples evaluated. A nuclear staining pattern was observed in 8% of NHL cases overall and 1.8% of DLBCL. Interestingly, the lymphoblastic T-cell (LBT) subtype had the highest percentage of nuclear staining with 40% of the lymphomas showing nuclear overexpression. As summarized in Table 1, we have established that PRMT5 exhibits variable overexpression in all subtypes of canine lymphoma.

**Table 1:** PRMT5 immunohistochemistry of canine lymphoma tissue microarrays.

Histology	Cytoplasmic staining			Nuclear staining	
	Strong	Weak	Negative	Yes	No
**Peripheral T-cell**	12	34	2	11	37
(n = 58)	25.00%	70.80%	4.20%	22.90%	77.10%
**Burkitt/Burkitt-like**	3	3	0	0	6
(n = 6)	50.00%	50.00%	0.00%	0.00%	100.00%
**Diffuse large B-cell**	82	84	2	3	165
(n = 168)	48.80%	50.00%	1.20%	1.80%	98.20%
**Follicular**	2	1	0	0	3
(n = 3)	66.70%	33.30%	0.00%	0.00%	100.00%
**Lymphoblastic T-cell**	2	7	1	4	6
(n = 10)	20.00%	70.00%	10.00%	40.00%	60.00%
**Marginal zone**	31	27	4	3	59
(n = 62)	50.00%	43.50%	6.50%	4.80%	95.20%
**Small cell lymphocytic**	5	15	2	4	18
(n = 22)	22.70%	68.10%	9.00%	18.10%	81.80%
**T-zone**	6	12	0	2	16
(n = 18)	33.30%	66.70%	0.00%	11.10%	88.90%
**Normal/hyperplastic**	0	29	11	2	38
(n = 40)	0.00%	72.50%	27.50%	5.00%	95.00%
**Total lymphoma**	143	183	11	27	310
(n = 337)	42.40%	54.30%	3.30%	8.00%	92.00%

Using qRT-PCR, we evaluated the relative abundance of *PRMT5* and *MYC* transcripts in canine primary lymph node biopsies (mRNA primers listed in S1 Table). In correlation with our TMA results, we observed high overexpression of PRMT5 in primary B-cell lymphomas compared to canine normal donor (ND) tissue (*p < 0*.*01*). Additionally, the proto-oncogene *MYC*, shown to be tightly linked with PRMT5 activity driving cellular proliferation and survival [35], was found to be overexpressed in primary B-cell lymphomas compared to canine ND tissue (*p < 0*.*05*) (Fig 1B). To validate these findings, we evaluated the protein expression of PRMT5 and MYC in the same lymphoid biopsy samples from Fig 1B. Compared to ND tissue, all B-cell and one of two T-cell lymphomas exhibit higher protein expression of MYC and PRMT5 (Fig 1C). Next, we evaluated the PRMT5 and MYC protein expression in three canine lymphoma-derived cell lines (17–71, CLBL-1, and OSW). Compared to ND fractioned B- and T-lymphocyte subsets in peripheral blood, all canine lymphoma cell lines showed higher levels of MYC and PRMT5 protein (Fig 1D).

### Inhibition of PRMT5 results in enhanced cell death and reduced growth of canine lymphoma cell lines and primary samples

**Fig 2:**
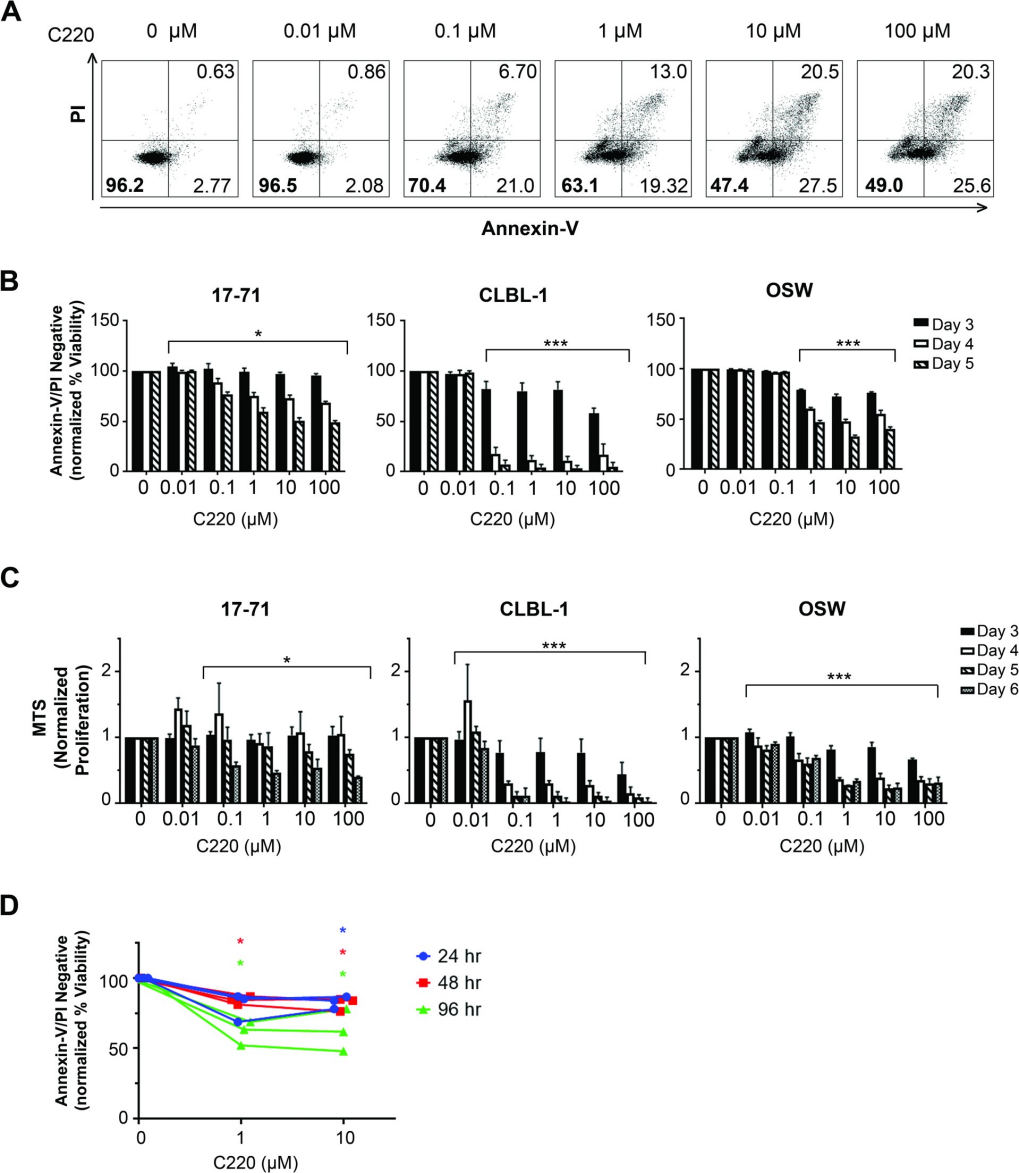
Inhibition of PRMT5 results in enhanced cell death and reduced growth of canine lymphoma cell lines and primary samples. (A) Representative flow cytometry plots of cell line 17–71 treated with the PRMT5 inhibitor (C220) for 5 days demonstrating staining for early (Annexin-V+/PI-) and late apoptotic (Annexin-V+/PI+) cells. Cell viability was quantified by the percentage of Annexin-V/PI double negative staining. The percentage of viable, non-apoptotic cells is indicated in the lower left quadrant. (B) Canine lymphoma cell lines were treated with log10 increasing concentrations of C220 and cell viability was measured at day 3, 4, and 5. The beta mixed regression model supported significantly decreasing linear trends between viability and day all cell lines (17–71: *p < 0.*04* for each concentration, CLBL-1: ***p < 0.*001* for concentrations ≥ 0.1 μM, and OSW: ***p < 0.*001* for concentrations ≥ 1 μM). (C) Canine lymphoma cell lines were treated with log10 increasing concentrations of C220 and viable cell proliferation was measured at day 3, 4, 5, and 6 by the MTS colorimetric assay for metabolic activity. For 17–71, the linear mixed model supported a significantly decreasing linear trend between MTS absorbance and day (17–71: *p < 0.*012* for concentrations ≥ 0.1 μM). For cell lines CLBL-1 and OSW the main effect of both log10 treatment concentration and day are both estimated to decrease MTS absorbance by 0.12 units (CLBL-1: *p <* 0.001) and 0.09 (OSW: *p <* 0.001) for every one unit increase. (D) Induction of apoptosis in primary lymphoma samples treated ex-vivo (n = 3 dogs per condition) indicated by a statistically significant decrease in viability compared to the normalized vehicle control (*p < 0.*05*, two-tailed Student’s t-test). For all graphs data are shown relative to the 0 nM DMSO control and are the averages of three or more biological replicate experiments. Error bars show mean ± SEM and respective p-values are indicated (*p < 0.*0*5, **p < 0.*01*, ***p < 0.*001*).

Pharmacological inhibition and genetic deletion of PRMT5 in human hematopoietic and solid tumor cell lines has been reported to lead to growth arrest and tumor cell death [28,31–34]. To determine whether PRMT5 activity is vital to growth and survival of canine lymphoma cells, we treated 17–71, CLBCL-1, and OSW cell lines with the highly selective PRMT5 inhibitor C220 and evaluated cellular viability by Annexin-V/PI staining with flow cytometry (Fig 2A and 2B) and viable cell proliferation by MTS (Fig 2C). For all cell lines, the beta mixed regression model showed significantly decreasing linear trends between viability and day (17–71: *p < 0*.*04* for each concentration, CLBL-1: *p < 0*.*001* for concentrations ≥ 0.1 μM, and OSW: *p < 0*.*001* for concentrations ≥ 1 μM) and between viability and log10 treatment concentration (17–71: *p < 0*.*011* for each day, CLBL-1: *p < 0*.*001* for days 4 and 5, and OSW: *p < 0*.*001* for each day) (Fig 2B).

For cell line 17–71, the linear mixed model supported a significantly decreasing linear trend between viable cell proliferation (MTS absorbance) and day (17–71: *p < 0*.*012* for concentrations ≥ 0.1 μM) and between viable cell proliferation and log10 treatment concentration (17–71: *p < 0*.*019* for days 4, 5, and 6) (Fig 2C). For cell lines CLBL-1 and OSW, the linear mixed model did not support and interaction effect between day and treatment concentration, rather the main effect of both log10 treatment concentration and day are both estimated to decreased MTS absorbance by 0.12 units (CLBL-1: *p <* 0.001) and 0.09 (OSW: *p <* 0.001) for every one unit increase. Notably, cell line CLBL-1 showed remarkable anti-proliferative activity (IC50 < 0.1 μM) after four days in culture as compared to the vehicle control. To validate these findings, we evaluated the effects of PRMT5 inhibition on primary canine lymphoma samples after 24, 48, and 96 hours (S2 Table). Ex-vivo treatment of primary lymphoma samples confirms the cytotoxic effects of PRMT5 inhibition with induction of apoptosis at each pair wise comparison to the control treatment condition (*p < 0*.*05*) (Fig 2D).

### Selectivity of PRMT5 inhibition with C220

**Fig 3:**
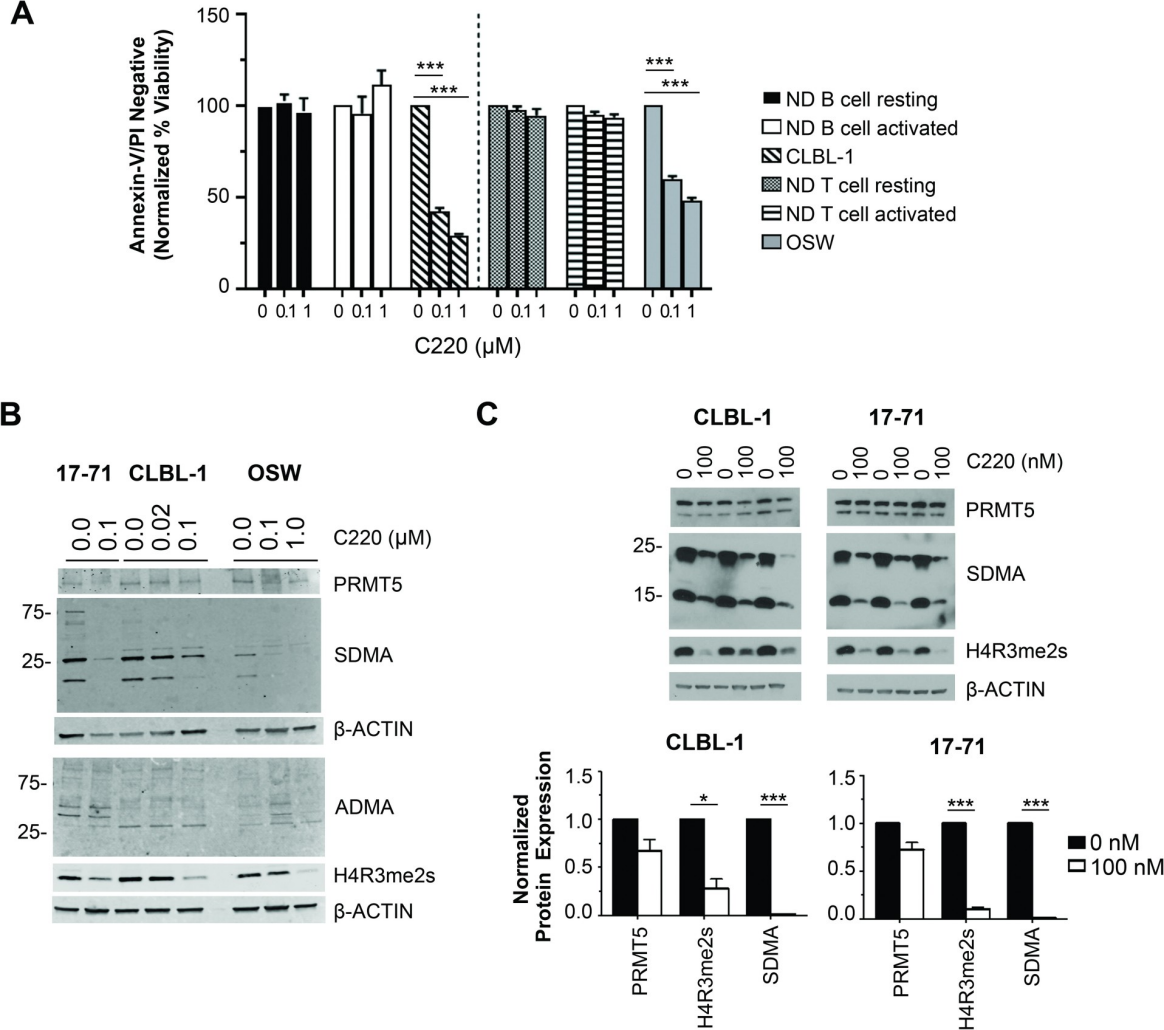
Selectivity of PRMT5 inhibition with C220. (A) PRMT5 inhibition is selectively toxic to canine lymphoma cells and not resting or activated ND B-cells or T-cells as determined by percent of viable cells over a 96-hour incubation. There are no statistically significant differences in predicted viabilities between any pairwise comparison of treatment concentration within the ND resting and activated B-cells or T-cells; whereas, the viability of the lymphoma cell lines (CLBL-1 and OSW) significantly decreased as treatment concentration increased (***p < 0.*001* for each pairwise comparison to vehicle control). (B) Cell lines 17–71 (day 4) and CLBL-1 (day 2) and OSW (day 3) were treated at the indicated concentration of C220 (μM). Whole cell lysates were immunoblotted for the proteins indicated. Western blots were detected using Licor IRDye conjugated secondary antibodies and the Licor Odyssey imaging system. For all cell lines, effects of PRMT5 inhibition with C220 treatment on biomarkers of PRMT5 activity shows a dose-dependent decrease in global marks of symmetric di-methylarginine (SDMA) and symmetric di-methylation of histone H4 arginine-3 (H4R3me2s) with a minimal effect to PRMT5 or global asymmetric di-methylarginine (ADMA). (C) Immunoblots and quantification of three biological replicate experiments, showing attenuated SDMA and H4R3me2s in 17–71 (day 4) and CLBL-1 (day 2) after treatment with 100 nM C220, with no significant decreased to PRMT5 expression. PRMT5, SDMA, and H4R3me2s with B-actin were ran on separate gels. Immunoblots were detected using film and HRP conjugated secondary antibodies. Protein expression was quantified by the ratio of target protein to loading control then normalized to the 0 nM DMSO control. Statistical significance evaluated with a paired two-tailed Student’s t-test compared to normalized vehicle control. For all graphs data are shown relative to the 0 nM DMSO control and are the averages of three or more biological replicate experiments. Error bars show mean ± SEM and p-values are indicated as such (*p < 0.*05*, *p < 0.*01*, ***p < 0.*001*).

Considering the low PRMT5 expression in canine ND lymph node samples and biologic role of PRMT5 in normal hematopoiesis, we sought to evaluate whether PRMT5 inhibition is selectively toxic to malignant cells and not normal cellular counterparts. Isolated B- and T-lymphocytes from the peripheral blood of canine NDs were treated with C220 for 96 hours with and without stimulation (isolation and stimulation procedure detailed in S1 Text). Cell viability was measured by Annexin-V/PI staining and flow cytometry. While PRMT5 inhibition showed dose-dependent anti-tumor activity in canine lymphoma cell lines, there was no significant effect on the viability of normal canine resting or activated B- or T-cells in culture, indicating that pharmacologic inhibition of PRMT5 using the small molecule inhibitor (C220) is selectively toxic to malignant cells (*p < 0*.*001*) (Fig 3A).

To establish whether the observed tumor cell death was associated with selective inhibition of PRMT5’s symmetric dimethyl transferase activity, we performed western blot analysis of protein extracts using anti-sera specific for symmetric and asymmetric dimethyl arginine. Global (SDMA and ADMA) and histone targeted (H4R3me2s) methylation marks on arginine residues were evaluated after pharmacologic inhibition of PRMT5 with compound C220 (complete materials and methods detailed in the S1 Text). Similar to human lymphoma, treatment of canine lymphoma cell lines showed a substantial decrease in global SDMA and H4R3me2s with minimal effect to PRMT5 expression or asymmetric dimethyl arginine (ADMA) as expected with selective inhibition of PRMT5 (Fig 3B and 3C).

### Changes to chromatin state with PRMT5 inhibition

**Fig 4:**
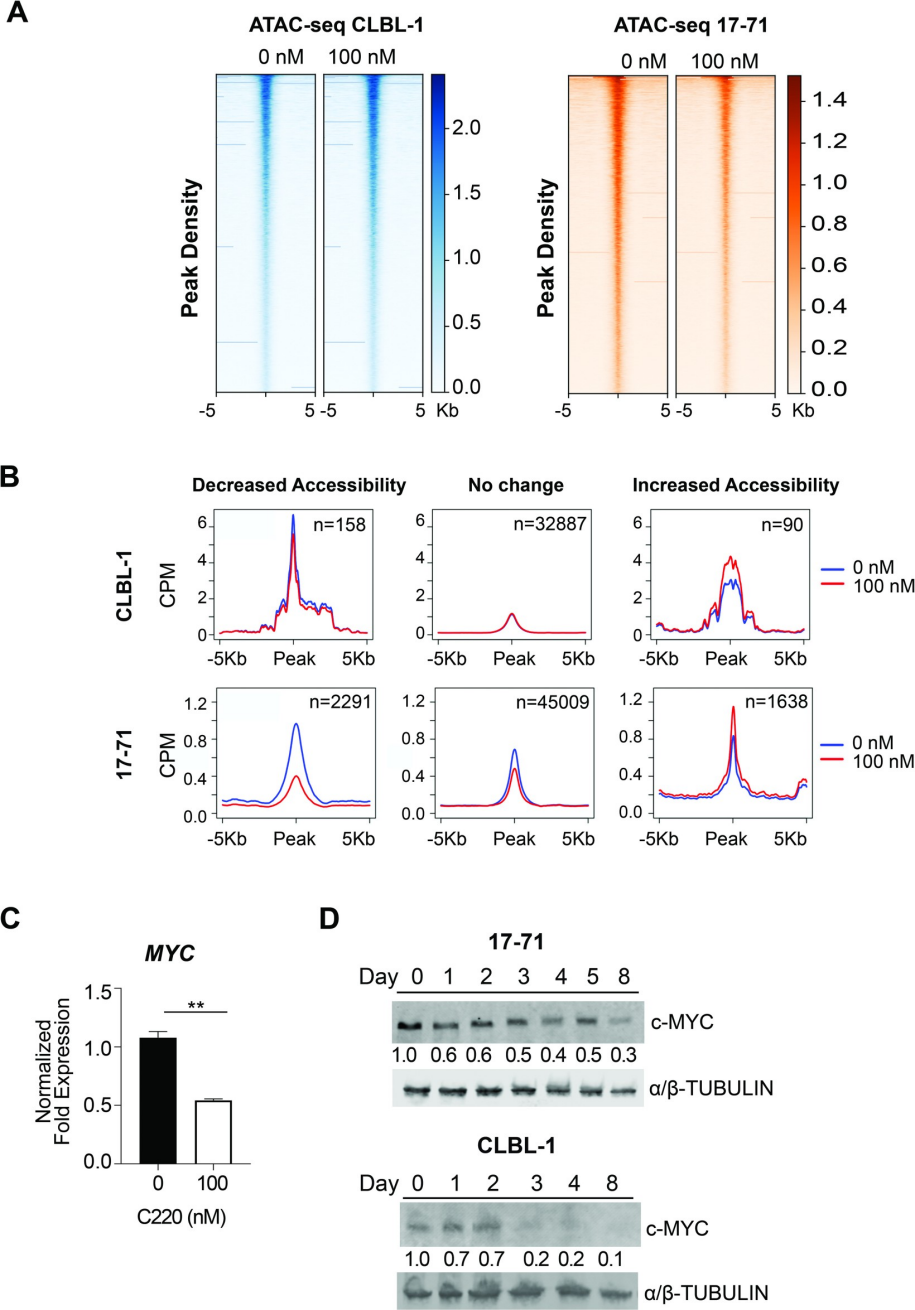
Changes to chromatin state with PRMT5 inhibition. The B-cell lymphoma cell lines 17–71 (day 4) and CLBL-1 (day 2) were treated with 100 nM of C220. Cells were harvested and nucleic DNA was extracted and processed as indicated in the methods section. ATAC-seq was performed using the HiSeq Illumina platform. (A) Heatmap of the top 20,000 ATAC-seq peak densities within 5Kb upstream and downstream of the peak center in descending order, shows no global change to chromatin accessibility after PRMT5 inhibition. Representative of biological triplicate experiments. (B) ATAC-seq normalized read counts (as counts per million (CPM)) within 5Kb downstream and 5Kb upstream of the peak center, showing decreased, unchanged, and increased accessibility upon C220 treatment. At a FDR of 10%, more genomic sites show decreased accessibility than increased. (C) qRT-PCR analysis confirmed a decrease in *MYC* transcript in CLBL-1 (day 2) after treatment with C220 (100 nM) compared to the 0 nM DMSO control (**p < 0.*01*). Data is an average of 2 biological replicate experiments analyzed using an unpaired, two-tailed Student’s t-test. (D) Immunoblot analysis indicates a time dependent decrease in MYC protein after treatment with C220 in 17–71 (100 nM) and CLBL-1 (20 nM). Immunoblots were detected with fluorescently labeled Licor IRDye secondary antibodies. Protein expression was quantified by the ratio of target protein to loading control and normalized to Day 0.

Given the role of PRMT5 to modulate transcriptional gene silencing via writer activity associated with repressive methylation marks on histone tails, we sought to characterize changes in whole-genome chromatin accessibility in the presence and absence of PRMT5 inhibition. Conformational changes to chromatin accessibility after PRMT5 inhibition could be either directly related to PRMT5’s repressive histone methylation or indirectly resulting from the attenuation of PRMT5’s methyltransferase enzymatic activity of numerous cytosolic and nuclear proteins. We utilized ATAC-seq to interrogate global changes to chromatin structure (euchromatin vs. heterochromatin) after PRMT5 inhibition [49]. Visualizing the top 20,000 peak densities (+/- 5Kb from the peak center) no dramatic change in accessibility was observed on a global scale (Fig 4A). Considering the lack of global change, we sought to identify specific sites of differential chromatin accessibility. Visualizing the average profile of ATAC-seq peaks after treatment with C220, indicated more sites with decreased accessibility than increased at FDR cutoff of 10% (Fig 4B). Applying an absolute fold change cutoff of 2, minimal changes were observed in cell line CLBL-1 treated for two days with 100 nM C220, whereas, cell line 17–71 treated for four days with 100 nM C220 showed 953 individual regions with changes to chromatin accessibility (FDR cutoff of 10%) (S3 Table). Of the 953 regions, 208 regions were within 5 kilobases of an annotated gene including 143 protein coding genes. Interestingly, of the nine known PRMT enzymes, only PRMT1, PRMT5, and PRMT9 showed an accessibility signal at the transcription start site. As expected, the accessibility of each PRMT did not change with selective inhibition of PRMT5’s enzymatic activity (S3 Table).

Of particular interest, *MYC* was among the top three differentially accessible chromatin sites, showing statistically significant decreased accessibility (Fold Change = -2.48, p = 1.07E-07, FDR = 0.1%) (S3 Table). Given the oncogenic role of MYC in lymphomagenesis and an established PRMT5/MYC axis in human cancer, we sought to validate the functionality of the PRMT5/MYC axis in canine lymphoma [50]. To establish if the decreased accessibility of *MYC* after PRMT5 inhibition affects the expression of MYC transcript or protein, we preformed qRT-PCR and immunoblot analysis after treatment with C220. Indeed, *MYC* transcript was decreased in cell line CLBL-1 after a two day treatment with C220 (100 nM) compared to the DMSO (0 nM) treatment control (*p < 0*.*01*, biological duplicate experiment) (Fig 4C). Likewise, we observed a time-dependent decrease in MYC protein after PRMT5 inhibition in cell lines CLBL-1 (20 nM) and 17–71 (100 nM) (Fig 4D). Together this data confirms the transcriptional regulation of *MYC* by selective inhibition of PRMT5.

### PRMT5 inhibition drives global transcriptomic changes in canine lymphoma

**Fig 5:**
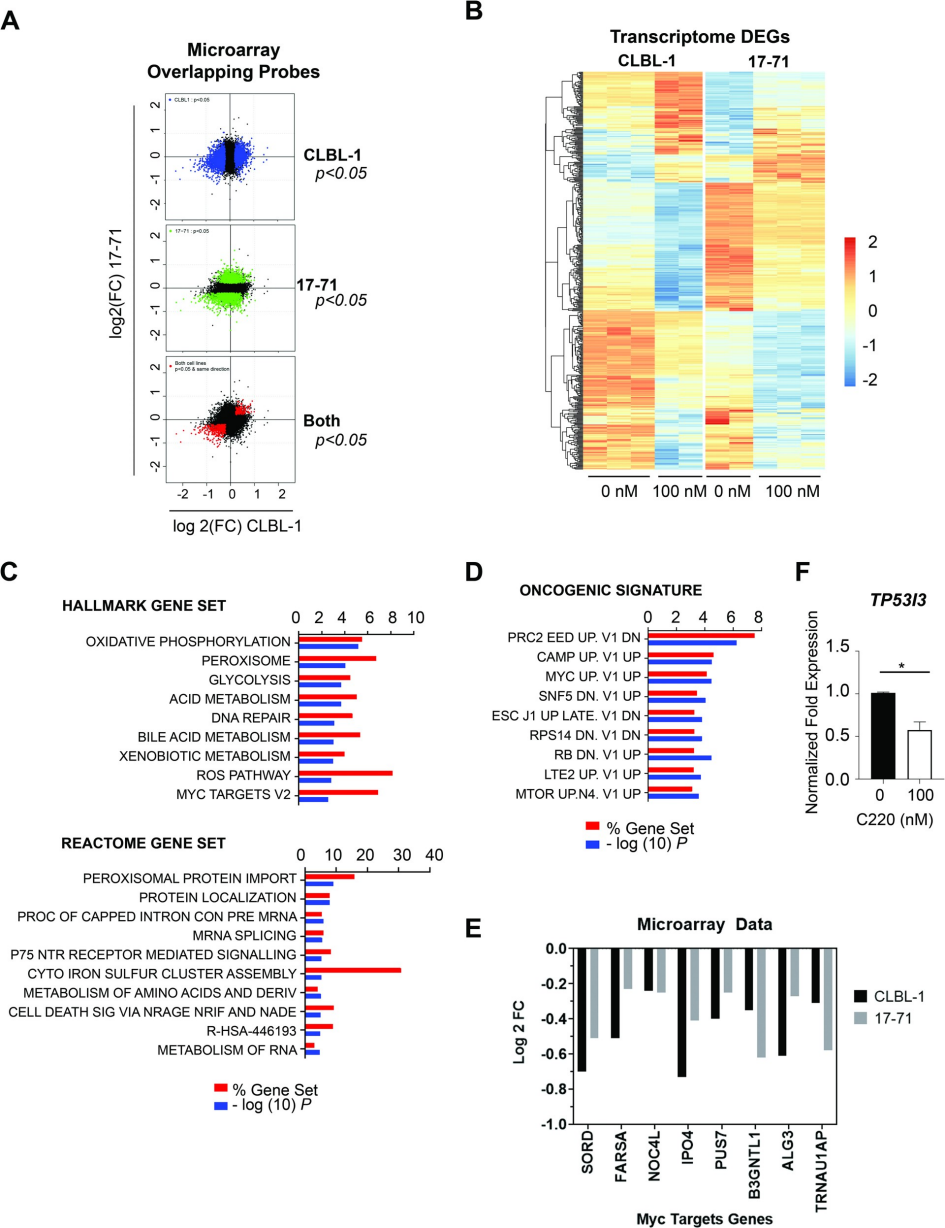
PRMT5 inhibition drives global transcriptomic changes in canine lymphoma. (A) Canine cell lines were treated with 100 nM of C220 for 2 days (CLBL-1) and 4 days (17–71) (biological triplicate experiments). Total RNA was processed as indicated (S1 Text). The Affymetrix canine gene chip microarray was used to identify differentially expressed transcripts after PRMT5 inhibition. Common treatment effect identified in both cell lines using p-value cutoff of 0.05 and same direction of change revealed 495 differentially expressed genes after PRMT5 inhibition in both cell lines. (B) Heatmap illustrating more genes downregulated than upregulated at the transcript level with PRMT5 inhibition. (C) Overlapping gene sets between our identified 495 DEGs and the Molecular Signatures Database (MSigDB) gene sets (Hallmark and Canonical Pathway: Reactome) (top 10 gene sets with FDR q value < 0.05). (D) Overlapping gene sets between the 290 downregulated DEGs after PRMT5 inhibition and the Molecular Signatures Database (MSigDB) gene sets (Oncogenic Signatures) (top 10 gene sets with FDR q value < 0.05). (E) Microarray data set identified significantly decreased MYC target genes with inhibition of PRMT5. Contained within the Hallmark gene set MYC Targets V2 (*SORD*, *FARSA*, *NOC4L*, and *IPO4*). Contained within the Oncogenic Signature gene set MYC UP V1 UP (*SORD*, *IPO4*, *NOC4L*, *PUS7*, *B3GNTL1*, *ALG3*, and *TRNAU1AP*). *SORD* (CLBL-1: *p <* 0.001, 17–71: *p <* 0.01), *FARSA* (CLBL-1: *p =* 0.001, 17–71: *p <* 0.05), *NOC4L* (CLBL-1: *p <* 0.05, 17–71: *p <* 0.05), *IPO4* (CLBL-1: *p <* 0.001, 17–71: *p <* 0.05), *PUS7* (CLBL-1: *p <* 0.01, 17–71: *p <* 0.05), *B3GNTL1* (CLBL-1: *p <* 0.05, 17–71: *p <* 0.01), *ALG3* (CLBL-1: *p <* 0.01, 17–71: *p <* 0.05), *TRNAU1AP* (CLBL-1: *p <* 0.01, 17–71: *p <* 0.05). (F) qRT-PCR analysis of *TP3I3* confirmed a decrease in transcript in CLBL-1 after a two day treatment with C220 (100 nM) compared to the 0 nM DMSO control (*p < 0.*05*). Data is an average of 2 biological replicate experiments analyzed using an unpaired, two-tailed Student’s t-test.

To further evaluate the effects of PRMT5 inhibition on canine lymphoma, we examined whole-transcriptome changes in the gene expression profile using an Affymetrix canine gene chip microarray (Affymetrix GeneChip CanGene 1.0 ST array) (Thermo Fisher Scientific, Waltham, MA). We identified 4395 and 2728 differentially expressed genes (DEGs) with a p-value cutoff of 0.05 upon C220 treatment in canine lymphoma cell lines 17–71 and CLBL-1, respectively. To identify a common treatment effect in both cell lines, a meta gene signature consisting of 495 probes was determined by taking the intersection of the DEGs in the same direction of change (p-value cut off of 0.05) (Fig 5A). Visualizing the meta gene signature heatmap, more genes show decreased rather than increased expression with PRMT5 inhibition (Fig 5B).

To establish the relevance of the resulting DEGs to known PRMT5 biology, Molecular Signatures Database (MSigDB) was used to compute the overlap between our 495 identified DEGs and curated gene sets in MSigDB. The significantly overlapping gene sets highlighted the essential role of PRMT5 in metabolic processes, oxidative phosphorylation, peroxisome, mRNA splicing, and DNA repair (top 10 gene sets with FDR q value < 0.05) (Fig 5C). Consistent with our ATAC-sequencing results showing decreased chromatin accessibility of *MYC* after PRMT5 inhibition, the Hallmark gene set MYC Targets V2 showed significant overlap with our list of DEGs after PRMT5 inhibition in the canine microarray data set (*p = 0*.*00275*, FDR q value < 0.05) and contained the four MYC target genes (*SORD*, *FARSA*, *NOC4L*, *IPO4*)(S4 Table and Fig 5E).

To elucidate the mechanism of PRMT5 inhibitor-mediated cell death in canine lymphoma, we performed a separate gene set overlap analysis between the 290 down-regulated DEGs after PRMT5 inhibition and MSigDB Oncogenic Signatures (top 10 gene sets with FDR q value < 0.05) (Fig 5D). This analysis overlapped significantly with gene expression changes after knock down of EED (a component of PRC2), genes up-regulated in cells overexpressing MYC, genes up-regulated after knock down of SNF5 (a core member of the SWI/SNF chromatin remodeling complex), and genes up-regulated after RB1 knock down (a key tumor suppressor). These results suggest that inhibition of PRMT5 in canine lymphoma can attenuate oncogenic MYC signaling while reactivating the RB tumor suppressor pathway and PRC2 silencing, an established mechanism of human lymphoma cell death after PRMT5 inhibition [37]. To establish the validity of our list of DEGs from the Microarray, qRT-PCR was performed. In correlation with our microarray results, we observed a decrease in *TP53I3* after a two day treatment of cell line CLBL-1 with 100 nM C220 (*p < 0*.*05*, biological duplicate experiment) (Fig 5F). Taken together, this whole-genome pathway interrogation demonstrates the feasibility of this approach for investigating mechanisms of PRMT5 inhibitor-mediated cell death in canine lymphoma.

## Discussion

Here, we have assessed the expression of PRMT5 in a large sample of histopathologically distinct subtypes of canine lymphoma. DLBCL and MZL are the most common B-cell lymphomas in the dog and PTCL is the most common T-cell lymphoma whereas other histologic subtypes are less common as reflected in the subset of this TMA. Thus, conclusions comparing relative PRMT5 expression and cellular localization in less common histologic subtypes of lymphoma must be made with caution. While PRMT5 was expressed in all tumors, the level of expression was variable, with over 42% of canine lymphomas showing strong expression based on immunohistochemical staining. PRMT5 immunoreactivity was mainly confined to the cytoplasm; however, nuclear staining was observed in 8% of all samples. Markedly, strong cytoplasmic expression of PRMT5 was common in DLBCL and nuclear staining was most common in LBT, occurring in approximately 40% of these tumors. PRMT5 has unique, pleiotropic regulatory roles in the cytoplasm and nucleus of cancer cells depending on the tissue or cell type in which it is expressed. Evidence supports strong nuclear staining of PRMT5 to be associated with aggressive behavior and poor prognosis in patients diagnosed with high grade glioma and neuroblastoma cancers [51,52]. Conversely, other studies report nuclear localization of PRMT5 to be associated with reduced cell growth and better patient outcomes in breast cancer and prostate cancer where nuclear exclusion signals have been identified [53–55]. In human lung cancers, cytoplasmic localization of PRMT5 was found to correlate with high-grade tumors, whereas PRMT5 nuclear localization was more frequent in well-differentiated carcinoid tumors [29]. Consequently, if PRMT5 expression is to be used as a prognostic indicator for response to targeted therapies directed at PRMT5, cellular localization should be evaluated as the functional role of this enzyme is almost certainly dependent on the nature of tissue and nuclear/cytoplasmic expression.

In our studies, the PRMT5 inhibition in primary canine lymphoma samples and cell lines showed significant anti-tumor effects. Similar to human lymphoma, the three cell lines tested showed a range of sensitivity to PRMT5 inhibition. The reason for cell line CLBL-1’s marked sensitivity to PRMT5 inhibition is likely genomic based on known duplications of tumor suppressor gene (PTEN) and duplication of MYC. We, and others, have found that MYC over-expression is supported by PRMT5 [35,56], thus it is plausible that MYC overexpression represents a therapeutic vulnerability to PRMT5 targeted therapy.

PRMT5 inhibition demonstrated limited toxicity to normal canine resting and activated B- and T-lymphocytes. To mitigate potential risk of toxicity to normal cells, SDMA is being evaluated as a biomarker of PRMT5 activity in human clinical trials evaluating the PRMT5 inhibitors compounds (JNJ-64619178, GSK3326595, and PRT543) in patients with relapsed/refractory non-Hodgkin lymphoma or advanced solid tumors (NCT03573310, NCT02783300, and NCT03886831). The ability to obtain serial tumor samples to assess treatment effects and develop relevant biomarkers represents an advantage of the canine spontaneous lymphoma model for drug development as compared to small rodent models. Establishing biomarkers of therapeutic response to PRMT5 inhibitions has valuable clinical implications. Furthermore, the spontaneous canine lymphoma model is clinically advantageous as it recapitulates the heterogeneity encountered in human lymphoma.

Since PRMT5 is a known epigenetic regulator involved in transcriptional silencing of genes due to repressive methylation marks on histone tails, we expected that differentially expressed transcripts would correlate with regional changes to chromatin accessibility leading to a restoration of the normal B-cell epigenetic landscape; however, this direct correlation was not observed. The identified genomic regions with changes in chromatin state observed in our ATAC-seq data set did not overlap with the majority of DEGs in our microarray data set. These findings suggest that in the context of canine lymphoma, the primary mechanism of cell death with PRMT5 inhibition may not be due primarily to changes in epigenetic histone methylation, but perhaps from methylation of other cellular proteins including spliceosome complex proteins as demonstrated by the list of differentially expressed gene transcripts (S4 Table and Fig 5C). Future studies are needed to determine the exact mechanism of PRMT5 inhibitor-mediated cell death and should incorporate RNA-sequencing with analysis of alternatively spliced variants. Given the dual epigenetic role of PRMT5 to promote transcriptional gene silencing (H2A, H3, and H4) or to promote euchromatic states poised for transcriptional activation (H3R2), additional studies should incorporate other technological advances such as Cleavage Under Targets and Tagmentation (CUT&Tag) to investigate protein-chromatin interactions and genomic localization of PRMT5 specific histone modifications. Furthermore, our ATAC-seq studies were performed on canine cell lines and thus, the chromatin landscape is likely to be significantly different than that seen in primary tumors. Future chromatin accessibility profiling should be performed on primary tissue using the optimized Omni-ATAC sequencing protocol with improved signaling-to-background ratio and increased number of genomic hot spots [57,58].

Here we report changes in chromatin state and whole-transcriptome profiles showing commonalities and distinctions between human and canine lymphomas coordinated by aberrant PRMT5 expression. Our data demonstrated a functional PRMT5/MYC axis in canine lymphoma, where pharmacologic inhibition of PRMT5 leads to reduced *MYC* expression resulting in the downregulation of MYC target genes including *SORD*, *FARSA*, *NOC4L*, and *IPO4*. The connection between MYC and PRMT5 is well supported in human lymphomagenesis, where PRMT5 is required for neoplastic growth and is driven by the oncogenic driver MYC [27]. Data from our lab has previously demonstrated that PRMT5 promotes the overexpression of MYC by directly inhibiting regulatory miRNAs and by supporting constitutive activation of WNT/beta-catenin signaling that drives AKT activity and the beta-catenin target gene expression of *MYC* [56,59]. Furthermore, it has been shown that during human lymphomagenesis, MYC regulates pre-messenger-RNA splicing fidelity by directly upregulating the translation of PRMT5, and other core small nuclear ribonucleoprotein assembly genes [35].

## Conclusion

In human cancers, PRMT5 is a known driver of malignant transformation and oncogenesis [27,33]. Many studies have shown that attenuating the activity of PRMT5 with direct pharmacological inhibition or genetic knockout results in malignant cell death and reduced growth. Although high PRMT5 expression is associated with increased cellular proliferation [60], the mechanisms by which this occurs are only partially understood as PRMT5 has many context-dependent biological roles. Understanding the exact mechanism of PRMT5 inhibitor-mediated cell death is critical to determining its potential as a therapeutic target. Although the safety and efficacy of the use of PRMT5 inhibitors for the treatment of human lymphoma is currently being established in ongoing clinical trials, further preclinical investigation is warranted. Taken together, our data supports the continued use of the spontaneous canine lymphoma model to study the oncogenic role of PRMT5 and further develop PRMT5 inhibitors for the treatment of lymphoma. Given the evidence for overexpression of PRMT5 as a driver in human and lymphoma associated with malignant activity in canine lymphoma, this model represents an ideal large animal platform for continued investigation and potential veterinary-clinical trials evaluating the activity and efficacy of PRMT5 inhibitors for NHL.

## Materials and methods

Primary canine lymphoma cells and normal donor blood samples were collected from dogs after obtaining owner consent. Sample collection was approved by the Ohio State University Institutional Animal Care and Use Committee (IACUC) and College of Veterinary Medicine Clinical Research Committee (CRC). A complete description of materials and methods are provided (S1 Text).

### Immunohistochemistry

PRMT5 expression in canine lymphoma tissue microarrays (TMAs) was assessed by immunohistochemistry. The TMAs were reviewed by two observers (KAR and KS) including a board certified pathologist (KS). PRMT5 expression was evaluated in cytoplasmic and nuclear compartments in comparison to normal and/or hyperplastic lymph node tissues. Since PRMT5 staining within TMA cores involved entire tissue (was diffuse) and varied in intensity, it was evaluated semi-quantitatively for intensity only. In cytoplasmic compartment it was recorded as negative (lack of staining), low (weak staining) and high (strong staining). Since there was less variation in nuclear staining, it was evaluated on a two-tier scale and recorded as negative (lack of staining) or positive (presence of staining).

### Cell culture

Established canine lymphoid cell lines 17–71 (B-cell lymphoma) [61,62], CLBL-1 (DLBCL) [63], and OSW (PTCL) [64] were used in this study [65]. Cell lines and primary canine lymphoma cells were cultured in complete RPMI 1640 medium supplemented with 10% heat inactivated fetal bovine serum (FBS) (Gibco), 2 mM L-glutamine (Invitrogen, Carlsbad, CA), 100 units/ml of penicillin and streptomycin (Gibco),and cultured at 37°C in 5% CO_2_. Normal canine peripheral blood mononuclear cells (PBMCs) were cultured in the above conditions with 20% FBS.

### Cell viability and proliferation assays

Cell viability and apoptosis were assessed by staining with Annexin-V/PI (BD Biosciences, San Jose, CA) followed by flow cytometric analysis as described in the FITC Annexin-V Apoptosis Detection Kit I protocol (BD Pharmingen^TM^ Technical Data Sheet, material number 556547). The percentage of live, non-apoptotic cells was quantified by gating on Annexin-V/PI double negative cells. Cell growth and proliferation were measured using a colorimetric MTS Assay Kit (Abcam) according to the manufacturer instructions. Canine lymphoma cell lines and primary cells were treated with the small molecule PRMT5 inhibitor C220 (Prelude Therapeutics, Inc., Wilmington, DE) dissolved in DMSO.

### Transcriptome profiling and ATAC-sequencing

For whole-transcriptome profiling and ATAC-sequencing experiments, cell lines CLBL-1 and 17–71 were treated with 100 nM of C220. Cell line CLBL-1 was collected at day 2 and cell line 17–71 was collected at day 4, according to cell line sensitivity and biomarker analysis of global protein symmetric di-methylation and H4R3me2s. The microarray data (GSE155599) and ATAC-sequencing data (GSE156084) have been deposited in the NCBI Gene Expression Omnibus (SuperSeries Accession number: GSE156086).

### Statistical analysis

Viability data were analyzed using beta mixed models on the non-normalized viability outcome values. For cell line flow data, the model included baseline viability (0 μM DMSO control) and the interaction of day and treatment concentration as fixed effects, as well as random effects of day to take account of the correlations among observations obtained on the same day. For experiments with normal donor cells, the model included the interaction of cell status (activated, resting, control) and treatment concentration as fixed effects, as well as random effects of donor to take account of the correlation among observations from the same donor. Similarly, MTS data were analyzed using linear mixed models with absorbance as the response, while baseline absorbance (0 μM DMSO control) and the interaction of day and treatment concentration were included as fixed effects. Comparisons between concentrations, days, and linear trends across days were explored. For western blot quantification, band intensity was quantified using Image Studio (Version 5.2). Statistical analyses were performed using R (version 3.6.1) with the glmmTMB (version 0.2.3) and lme4 (version 1.1–21) packages, Microsoft Excel, and GraphPad Prism (Version 7.02). For all bar graphs, data are an average of three or more replicate experiments unless otherwise specified, normalized to DMSO control, and expressed as the mean ± standard error of the mean (SEM). The two-tailed Student’s t test was used to determine the level of statistical significance as indicated in the respective figures. P-values are denoted as such (*p < 0.*05*, **p < 0.*01*, ***p < 0.*001*).

## Supporting information

S1 Text Materials and methods. (DOCX)

S1 Table Quantitative real time RT-PCR primer and probe set. (DOCX)

S2 Table Demographics of canine lymphoma sample donors. (DOCX)

S3 Table ATAC-seq results. (XLSX)

S4 Table Microarray results. (XLSX)

S1 Raw images Original images for blots and gels. (TIF)
