# Supplementary material for: Validation of protein arginine methyltransferase 5 (PRMT5) as a candidate therapeutic target in the spontaneous canine model of non-Hodgkin lymphoma
Source: PLoS One. 2021 May 14;16(5):e0250839. doi: 10.1371/journal.pone.0250839 (PMC8121334; doi:10.1371/journal.pone.0250839)
Supplement: S1 Text — (DOCX) [file pone.0250839.s001.docx]

**Primary sample collection and processing**

Primary canine lymphoma cells and normal donor blood samples were collected from dogs after obtaining owner consent. Sample collection was approved by the Ohio State University Institutional Animal Care and Use Committee (IACUC) and College of Veterinary Medicine Clinical Research Committee (CRC). Cells obtained by fine needle aspiration of peripheral lymph nodes or extra-nodal masses were washed with sterile phosphate buffered saline (PBS) and immediately cultured or cryopreserved in freezing-media (10% DMSO in FBS). Biopsies of peripheral lymph nodes were immediately flash froze in liquid nitrogen and stored at -80°C then processed using the PARIS™ protein and RNA isolation kit (Life Technologies Cat# AM1921). Peripheral blood samples were collected into heparinized blood tubes and processed by density centrifugation, followed by either enrichment of B-cells using anti-canine CD21-PE antibody (Clone CA2.1D6) or T-cells using anti-canine CD5-PE (Clone YKIX322.3) and the Easy Sep PE Positive Selection Kit (StemCell Technologies, Vancouver-Canada) or by flow cytometry cell sorting (FACS) with staining for CD21-PE and CD5-FITC. Enrichment was confirmed by flow cytometry. Isolated B-cells obtained from normal donor blood samples were stimulated with 10 ug/ml of plate-bound anti-human IgM (MP Biomedical, Santa Ana, CA) and 15 ng/ml of recombinant human interleukin-4 (IL-4) (Invitrogen). Isolated T-cells were stimulated with 10 ug/ml of plate bound anti-CD3 (eBioscience, San Diego, CA) and 10 ug/ml of soluble anti-CD28 (eBioscience) or alternatively with 5 ug/ml of soluble PHA (Invitrogen) and 30 ng/ml recombinant human interleukin-21 (IL-21) (R&D Systems, Minneapolis, MN). Flow cytometric staining with Cell Proliferation Dye (Invitrogen) was used to confirm stimulation prior to downstream analysis. Following stimulation, cells were treated with C220 for the indicated concentrations and time.

## **Immunohistochemistry**

Immunohistochemical staining was performed on canine lymphoma tumor microarrays (TMAs) constructed from archived formalin-fixed paraffin-embedded blocks containing previously histologically subtyped canine nodal lymphoma tissue. TMA blocks were cut at a 4 µm thickness and sections were placed on positively charged glass slides. Slides with sections were then placed in a 60°C oven for one hour, cooled, deparaffinized, and rehydrated through xylenes and graded ethanol solutions to water. Slides were quenched for five minutes in a 3% hydrogen peroxide solution in water to block endogenous peroxidase activity. Slides then underwent heat-induced epitope retrieval employing Target Retrieval Solution (Dako, Carpinteria, CA) for 25 minutes at 96°C in a vegetable steamer (Black & Decker, Towson, MD) and cooled for 15 minutes. Slides were then placed on a Dako Autostainer Immunostaining System (Dako). All incubations were at room temperature. TMA slides were incubated with a primary rabbit polyclonal anti-PRMT5 antibody (Abcam, Cambridge, MA) at 1:200 dilution for 60 minutes. TMA slides were then incubated with a goat anti-rabbit secondary antibody at 1:1000 dilution for 60 minutes (Abcam). Staining was visualized with the Vulcan Fast Red chromogen (Biocare Medicals, Concord, CA). Slides were counterstained in Richard Allen hematoxylin (Thermo Scientific, Middletown, VA), dehydrated through graded ethanol solutions, cleared in xylene, and cover slipped. For each TMA, another slide was stained using the same protocol, but omitting the primary antibody [1].

**Immunoblot analysis**

Whole cell lysates were extracted in 150 nM NaCl RIPA Lysis buffer supplemented with Halt^TM^ Protease and Phosphatase Inhibitor Cocktail (Thermo Fisher Scientific) followed by SDS-page polyacrylamide gel electrophoresis and transfer to a polyvinylidene difluoride (PVDF) membrane for protein detection with the indicated primary antibodies: anti-SDMe-Arginine (Cat. #13222S), anti-ADMe-Arginine (Cat. #13522S), anti-MYC (Cat. #9402S), α/β-Tubulin (Cat. #2148S), β-Actin (Cat. #4970S) (Cell Signaling Technologies, Denvers, MA), anti-PRMT5 (Cat. #Sc-22132 or #Sc-376937) (Santa Cruz Biotechnology, CA), anti-H4R3 Symmetric Dimethyl (Cat. #A-3718) (Epigentek, Farmingdale, NY). Membranes were incubated overnight in primary antibodies at 4°C, washed with 0.1% Tween-20 in TBS, and detected with either film using secondary antibodies conjugated to HRP (Cat. #7074, Cat. # 7067) (Cell Signaling Technologies, Denver, MA) or detected with the Odyssey CLx imaging system using the appropriate Licor IRDye secondary antibodies: goat anti-rabbit IgG (Cat. #926-32211, Cat. #926-68071), goat anti-mouse IgG (Cat. #926-68070, Cat. # 926-32210), or donkey anti-goat IgG (Cat. #926-32214, Cat. #926-68074) (LI-COR Biosciences, Lincoln, NE).

**ATAC-Sequencing**

Genomic DNA was isolated from cellular nuclei and immediately resuspended in transposase reaction mix using the Nextera DNA Library Prep Kit (Illumina, San Diego, CA) followed by purification with the Qiagen Minlute Kit. Tagmented DNA was amplified using the Nextera Index Kit (Illumina) followed by an additional purification step with the Qiagen MinElute Kit as described elsewhere [2]. DNA integrity was interrogated using the Agilent 2100 Bioanalyzer (Agilent Technologies). Raw data were collected using the Illumina HiSeq sequencing platform. Sequencing reads were aligned to the canine reference genome CamFam3.1 using Bowtie2 v2.2.4 [3] with “-t -k 1 -p 8 --very-sensitive --no-discordant --no-mixed --reorder -X 2000” parameters. Broad peak calls were generated using Macs2 with “--format BAMPE -g hs -B --broad --nomodel --shift -100 --extsize 200” parameters and p-value cutoff of 10^-5^ [4]. Spearman correlation of read counts per 10 kilobase was generated using deepTools multiBamSummary and plotCorrelation functions, and outlier samples were eliminated. Bigwig files were generated using deepTools bamCoverage function with “--binSize 20 --smoothLength 60 --normalizeUsing CPM --ignoreForNormalization chrX chrM” parameters [5]. An absolute fold change of 2 and FDR cutoff of 10% were used to identify chromatic regions of differential accessibility.

**Transcriptome Profiling**

To measure mRNA levels of target genes, total RNA was isolated and real-time quantitative reverse transcription polymerase chain reaction (qRT-PCR) was carried out using the Applied Biosystems TaqMan assay and the indicated primers (S1 Table 1). To normalize mRNA expression, levels of 18S rRNA were measured in both control and test cell lines using 1X premixed primer/probe set (Applied Biosystems, Inc., Foster City, CA, USA). For whole transcriptome expression profiling, total RNA was extracted using the Total RNA Purification Kit (Norgen) and QIAshredder Columns (Qiagen). RNA integrity was interrogated using the Agilent 2100 Bioanalyzer (Agilent Technologies, Palo Alto, CA). A 100 ng aliquot of total RNA was linearly amplified and labeled using the Affymetrix WT Plus Reagent Kit (Thermo Fisher Scientific, Waltham, MA). Then 5.2 μg of fragmented and labeled ss-cDNA were hybridized to Affymetrix GeneChip CanGene 1.0 ST array for 16 hours at 45°C rotating at 60 rpm. Arrays were washed and stained using the Fluidics Station 450 and scanned using the GeneChip Scanner 3000 7G (Affymetrix). Arrays were normalized prior to gene expression analysis. Microarray data was analyzed using the robust multiarray averaging (RMA) method in the R statistical programming environMent version 3.1.2 (R Foundation for Statistical Computing, Vienna, Austria). Differential expression analysis was performed with the R *‘limma’* package and graphically visualization of heat maps was performed with the R basic plots and *‘pheatmap’*. 495 differentially expressed genes of the same directionality with p-values less than 0.05 were selected for downstream functional pathway analysis using Toppgene. Microarray results and Toppgene functional enrichment analysis are provided in (S4 Table). Molecular Signatures Database (MSigDB) was used to compute the overlaps between our identified DEGs and the flowing gene sets in MSigDB: Hallmark, Canonical Pathway: Reactome, and Oncogenic Signatures) (top 10 gene sets with FDR q value < 0.05) [6, 7].

**Supporting Information References**

1. Shilo K, Wu X, Sharma S, Welliver M, Duan W, Villalona-Calero M, et al. Cellular localization of protein arginine methyltransferase-5 correlates with grade of lung tumors. Diagn Pathol. 2013;8:201. Epub 2013/12/10. doi: 10.1186/1746-1596-8-201. PubMed PMID: 24326178; PubMed Central PMCID: PMCPMC3933389.

2. Buenrostro JD, Giresi PG, Zaba LC, Chang HY, Greenleaf WJ. Transposition of native chromatin for fast and sensitive epigenomic profiling of open chromatin, DNA-binding proteins and nucleosome position. Nat Methods. 2013;10(12):1213-8. Epub 2013/10/06. doi: 10.1038/nmeth.2688. PubMed PMID: 24097267; PubMed Central PMCID: PMCPMC3959825.

3. Langmead B, Salzberg SL. Fast gapped-read alignment with Bowtie 2. Nat Methods. 2012;9(4):357-9. Epub 2012/03/04. doi: 10.1038/nmeth.1923. PubMed PMID: 22388286; PubMed Central PMCID: PMCPMC3322381.

4. Zhang Y, Liu T, Meyer CA, Eeckhoute J, Johnson DS, Bernstein BE, et al. Model-based analysis of ChIP-Seq (MACS). Genome Biol. 2008;9(9):R137. Epub 2008/09/17. doi: 10.1186/gb-2008-9-9-r137. PubMed PMID: 18798982; PubMed Central PMCID: PMCPMC2592715.

5. Ramírez F, Dündar F, Diehl S, Grüning BA, Manke T. deepTools: a flexible platform for exploring deep-sequencing data. Nucleic Acids Res. 2014;42(Web Server issue):W187-91. Epub 2014/05/05. doi: 10.1093/nar/gku365. PubMed PMID: 24799436; PubMed Central PMCID: PMCPMC4086134.

6. Liberzon A, Birger C, Thorvaldsdóttir H, Ghandi M, Mesirov JP, Tamayo P. The Molecular Signatures Database (MSigDB) hallmark gene set collection. Cell Syst. 2015;1(6):417-25. doi: 10.1016/j.cels.2015.12.004. PubMed PMID: 26771021; PubMed Central PMCID: PMCPMC4707969.

7. Subramanian A, Tamayo P, Mootha VK, Mukherjee S, Ebert BL, Gillette MA, et al. Gene set enrichment analysis: a knowledge-based approach for interpreting genome-wide expression profiles. Proc Natl Acad Sci U S A. 2005;102(43):15545-50. Epub 2005/09/30. doi: 10.1073/pnas.0506580102. PubMed PMID: 16199517; PubMed Central PMCID: PMCPMC1239896.
