# Supplementary material for: Validation of protein arginine methyltransferase 5 (PRMT5) as a candidate therapeutic target in the spontaneous canine model of non-Hodgkin lymphoma
Source: PLoS One. 2021 May 14;16(5):e0250839. doi: 10.1371/journal.pone.0250839 (PMC8121334; doi:10.1371/journal.pone.0250839)
Supplement: S1 Table — (DOCX) [file pone.0250839.s002.docx]

**S1 Table. Quantitative real time RT-PCR primer and probe set.**

| **Name** | **Sequence** | **Probe** |
| --- | --- | --- |
| canine MYC-2 F | 5’-cacggaggagaatgacaagag-3' | 82 |
| canine MYC-2 R | 5’-caaagaagctccgtttcagc-3' | 82 |
| canine TP53I3-1-F | 5’-ggacccgaaaacctttacttg-3' | 10 |
| canine TP53I3-1-R | 5’-aggaggacttcgccctctac-3' | 10 |
