# Supplementary material for: Validation of protein arginine methyltransferase 5 (PRMT5) as a candidate therapeutic target in the spontaneous canine model of non-Hodgkin lymphoma
Source: PLoS One. 2021 May 14;16(5):e0250839. doi: 10.1371/journal.pone.0250839 (PMC8121334; doi:10.1371/journal.pone.0250839)
Supplement: S2 Table — (DOCX) [file pone.0250839.s003.docx]

**S2 Table. Demographics of canine lymphoma sample donors.** Supplemental patient information corresponding to the samples used in Fig 2D showing induction of apoptosis with PRMT5 inhibition. M = male, F = female, DLBCL = diffuse large B cell lymphoma, PTCL = peripheral T cell lymphoma.

| **Patient Signalment** | **Age**  **(years)** | **Sex** | **Lymphoma Subtype** | **Treatment Naïve** | **Treatment Time (hours)** |
| --- | --- | --- | --- | --- | --- |
| Boxer | 7 | M | PTCL | No | 24 |
| Mixed | 5 | M | Unknown | Yes | 24 |
| Basset Hound | 11 | M | DLBCL | Yes | 24 |
| Goldendoodle | 5 | M | DLBCL | Yes | 48 |
| Golden Retriever | 4 | M | DLBCL | Yes | 48 |
| Mixed | 6 | M | Unknown | No | 48 |
| Basset Hound | 10 | M | DLBCL | Yes | 96 |
| Mixed | 10 | M | Unknown | Yes | 96 |
| Boxer | 8 | F | PTCL | Yes | 96 |
